# Data on CUX1 isoforms in idiopathic pulmonary fibrosis lung and systemic sclerosis skin tissue sections

**DOI:** 10.1016/j.dib.2016.08.014

**Published:** 2016-08-10

**Authors:** Tetsurou Ikeda, Maria Fragiadaki, Xu Shi-wen, Markella Ponticos, Korsa Khan, Christopher Denton, Patricia Garcia, George Bou-Gharios, Akio Yamakawa, Chikao Morimoto, David Abraham

**Affiliations:** a Royal Free and University College Medical School, London, UK; b Imperial College School of Medicine, London, UK; c University of Tokyo, Institute of Medical Science, Tokyo, Japan

## Abstract

This data article contains complementary figures related to the research article entitled, “Transforming growth factor-β-induced CUX1 isoforms are associated with fibrosis in systemic sclerosis lung fibroblasts” (Ikeda et al. (2016) [2], http://dx.doi.org/10.1016/j.bbrep.2016.06.022), which presents that TGF-β increased CUX1 binding in the proximal promoter and enhancer of the *COL1A2* and regulated COL1. Further, in the scleroderma (SSc) lung and diffuse alveolar damage lung sections, CUX1 localized within the α- smooth muscle actin (α-SMA) positive cells (Fragiadaki et al., 2011) [1], “High doses of TGF-beta potently suppress type I collagen via the transcription factor CUX1” (Ikeda et al., 2016) [2]. Here we show that CUX1 isoforms are localized within α-smooth muscle actin-positive cells in SSc skin and idiopathic pulmonary fibrosis (IPF) lung tissue sections. In particular, at the granular and prickle cell layers in the SSc skin sections, CUX1 and α-SMA are co-localized. In addition, at the fibrotic loci in the IPF lung tissue sections, CUX1 localized within the α-smooth muscle actin (α-SMA) positive cells.

**Specifications Table**

**Table:** 

Subject area	*Biology*
More specific subject area	*Fibrosis*
Type of data	*Image*
How data was acquired	*Confocal microscopy (Zeiss Axioscope light microscope, Carl Zeiss)*
Data format	*Analysed*
Experimental factors	For immunohistochemistry, sections were pretreated with methanol, followed by antigen retrieval in heated 10-mM citrate buffer (pH 6).
Experimental features	The primary antibodies were CUX1 antibody (0.2 mg/ml) and the monoclonal anti-α-SMA clone, 1a Cy3-conjugated antibody (0.7 mg/ml). Sections were sequentially incubated with a 1/200 dilution of an Alexa 488 secondary antibody.
Data source location	*London, United Kingdom*
Data accessibility	*Data presented in this article*

**Value of the data**

• The data provides the evidence that CUX1 isoforms localise within α-smooth muscle actin-positive cells in SSc skin tissue sections, especially in the granular and prickle cell layers.
• The data provides the evidence that CUX1 isoforms localise within α-smooth muscle actin-positive cells at the fibrotic loci in idiopathic pulmonary fibrosis (IPF) lung tissue sections.
• The data could further help research on the function of CUX1 isoforms in fibrosis-related diseases.

## 1 Data

**Fig. 1:**
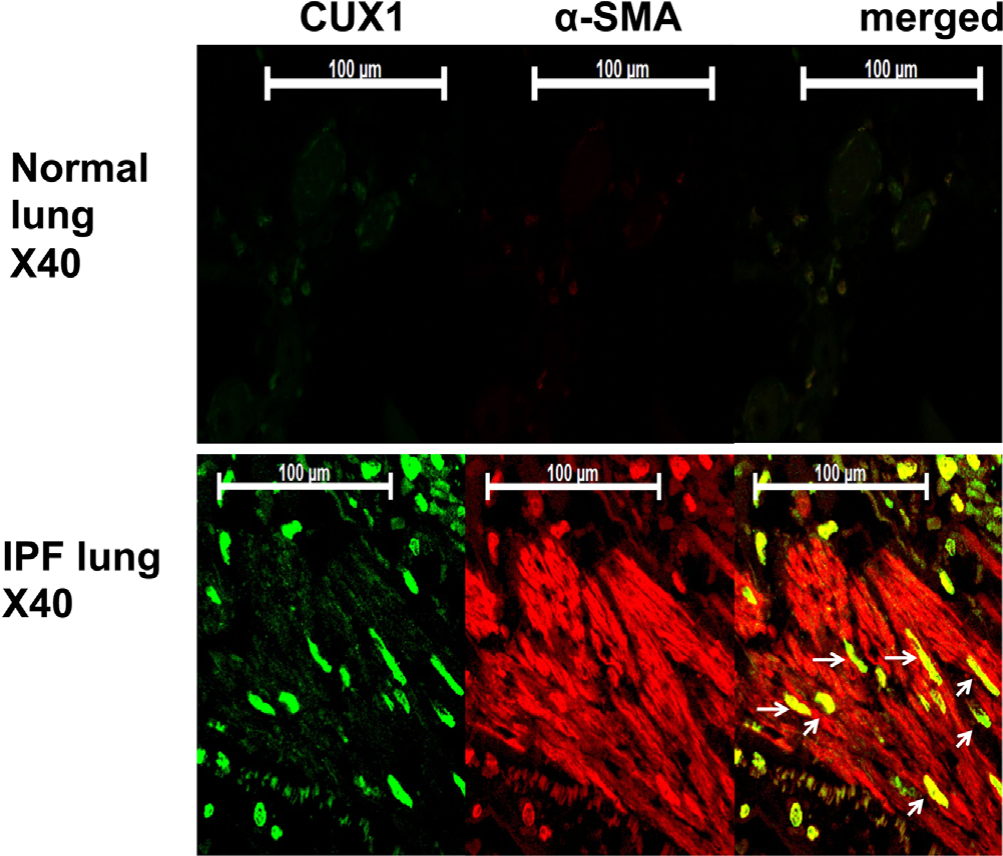
Idiopathic pulmonary fibrosis (IPF) lung tissue sections stained with antibodies against CUX1 and α-smooth muscle actin (SMA). The figure shows fibrotic loci that were stained by CUX1 and α-SMA antibodies. Alveolar cells around the loci were positive for CUX1 and α-SMA. CUX1 localised within α-SMA-positive cells. Normal lung sections were used as negative control for CUX1 and α-SMA.

**Fig. 2:**
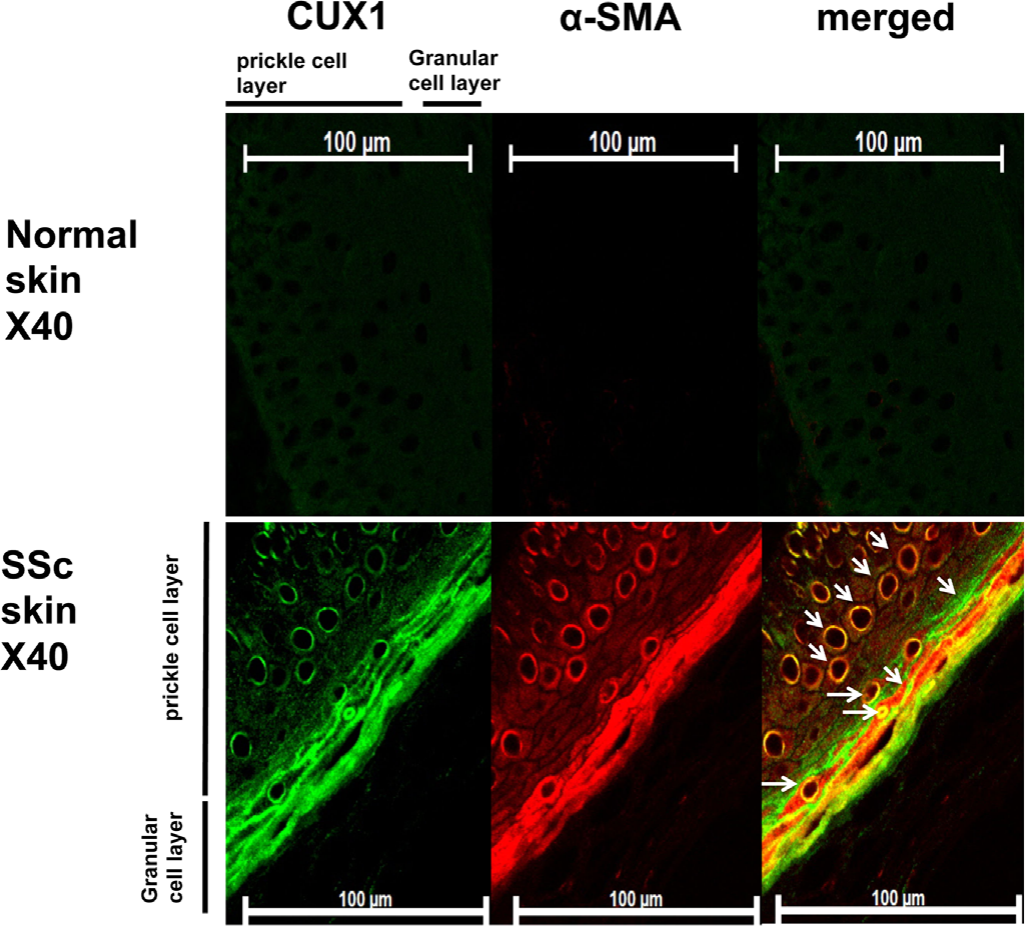
Systemic sclerosis (SSc) skin tissue sections stained with antibodies against CUX1 and α-smooth muscle actin (SMA). The figure shows epidermis that were stained by CUX1 and α-SMA antibodies. The granular and prickle cells in the epidermis were positive for CUX1 and α-SMA. CUX1 localised within α-SMA-positive cells. Normal skin sections were used as negative control for CUX1 and α-SMA.

Immunohistochemistry revealed the presence of α-SMA-positive fibrotic loci, which are characteristic in the lungs of patients with IPF and in the skin of patients with SSc. In the IPF tissue sections, CUX1 localised at alveolar cells and fibrotic loci. In addition, CUX1 localised within α-SMA-positive cells but was not observed in the normal lung section (Fig. 1). In the SSc skin tissue sections, CUX1 co‐localised with α-SMA at the granular cell layer, prickle cell layer and fibrotic loci in the epidermis. In addition, CUX1 localised within α-SMA-positive cells. These were not observed at the epidermis in the normal skin sections (Fig. 2).

## 2 Experimental design, materials and methods

For formalin-fixed, paraffin-embedded specimens, the tissues were fixed and hydrated as described previously [1], [2]. For immunohistochemistry, the sections were pretreated with methanol (VWR, Lutterworth, UK), followed by antigen retrieval in heated 10-mM citrate buffer (pH 6). The primary antibodies were CUX1 antibody (0.2 mg/ml) and the monoclonal anti-α-SMA clone, 1a Cy3-conjugated antibody (0.7 mg/ml). The sections were sequentially incubated with a 1/200 dilution of an Alexa 488 secondary antibody. The slides were viewed and photographed using a Zeiss Axioscope light microscope with Axiovision software.
