# Supplementary material for: Data on CUX1 isoforms in idiopathic pulmonary fibrosis lung and systemic sclerosis skin tissue sections
Source: Data Brief. 2016 Aug 10;8:1377–80. doi: 10.1016/j.dib.2016.08.014 (PMC4993856; doi:10.1016/j.dib.2016.08.014)
Supplement: Supplementary file 1 — Supplementary material [file mmc1.zip › coi_disclosure George Bou-Gharios.pdf]

ICMJE INTERNATIONAL COMMITTEE of MEDICAL JOURNAL EDITORS

SAVE

ICMJEForm for Disclosure of Potential Conflictsof Interest

Instructions

The purpose of thisform is to provide readersof your manuscript with information about your other interests that could influence how they receive and understand your work. The form is designed to be completed electronically and stored electronically. It contains programming that allowsappropriate data display. Each authorshouldsubmit aseparate form and is responsible for the accuracy andcompletenessof thesubmitted information. The form isinsix parts.

1. Identifying information.

2. The work underconsideration for publication.

Thissection asksfor information about the work that you havesubmitted for publication. The time frame for this reporting is that of the work itself, from the initial conception and planning to the present. The requested information isabout resources that you received, either directly or indirectly (viayour institution), to enable you to complete the work. Checking "No" means that you did the work without receiving any financial support from any third party -- that is, the work wassupported by funds from thesame institution that paysyour salary and that institution did not receive third-party funds with which to pay you. If you or your institution received funds from a third party tosupport the work, such asa government granting agency, charitable foundation or commercial sponsor, check "Yes".

3. Relevant financial activitiesoutside thesubmitted work.

Thissection asksabout your financial relationships with entities in the bio-medical arena that could be perceived to influence, or that give theappearance of potentially influencing, what you wrote in thesubmitted work. You should disclose interactions with ANYentity that could be considered broadly relevant to the work. For example, if your article isabout testing an epidermal growth factor receptor (EGFR)antagonist in lung cancer, you should report all associations with entities pursuing diagnostic or therapeuticstrategies in cancer in general, not just in thearea of EGFRorlung cancer.

Report all sources of revenue paid (or promised to be paid) directly to you or your institution on your behalf over the 36 months prior to submission of the work. Thisshould includeall monies from sources with relevance to thesubmitted work, not just monies from the entity that sponsored the research. Please note that your interactions with the work'ssponsor that are outside thesubmitted work should also be listed here. If there isany question, it is usually better to disclosea relationship than not to doso.

For grantsyou have received for work outside thesubmitted work, you should disclosesupport ONLY from entities that could be perceived to beaffected financially by the published work, such as drug companies, or foundationssupported by entities that could be perceived to havea financial stake in the outcome. Public fundingsources, such as government agencies, charitable foundations or academic institutions, need not be disclosed. For example, if a government agencysponsored astudy in which you have been involved and drugs were provided by a pharmaceutical company, you need only list the pharmaceutical company.

4. Intellectual Property.

Thissection asksabout patentsand copyrights, whether pending, issued, licensed and/or receiving royalties.

5. Relationships not covered above.

Use thissection to report other relationships or activities that readers could perceive to have influenced, or that give theappearance of potentially influencing, what you wrote in thesubmitted work.

Definitions.

Entity: government agency, foundation, commercial sponsor, academic institution, etc.
Grant: A grant from an entity, generally [but not always] paid to your organization
Personal Fees: Monies paid to you for services rendered, generally honoraria, royalties, or fees for consulting , lectures, speakers bureaus, expert testimony, employment, or other affiliations
Non-Financial Support: Examples include drugs/equipment supplied by the entity, travel paid by the entity, writing assistance, administrativesupport, etc.

Other: Anything not covered under the previous three boxes
Pending: The patent has been filed but not issued
Issued: The patent has been issued by theagency
Licensed: The patent has been licensed to an entity, whether earning royalties or not
Royalties: Fundsarecoming in to you or your institution due to your patent

INTERNATIONAL COMMITTEE of MEDICAL JOURNAL EDITORS

SAVE

ICMJEForm for Disclosure of Potential Conflictsof Interest

Section 1 Identifying Information

1. Given Name (First Name)
George

2. Surname (LastName)
Bou-Gharios

3. Date
27-March-2016

4. Are you the corresponding author ☒ No ☐

5. Manuscript Title
Transforming growth factor-β-induced CUX1 isoforms areassociated with fibrosis in systemicsclerosis lung fibroblasts

6. Manuscript Identifying Number (if you know it)

Section 2 The Work UnderConsideration for Publication

Did you or your institution **at any time** receive payment or services from a third party (government, commercial, private foundation, etc.) for any aspect of thesubmitted work (including but not limited to grants, data monitoring board, study design, manuscript preparation, statistical analysis,etc.)?

Are thereany relevant conflicts of interest? ☐ Yes ☒ No

ADD

Section 3 Relevant financial activitiesoutside thesubmitted work.

Place acheck in theappropriate boxes in the table to indicate whether you have financial relationships (regardlessof amount of compensation) with entitiesas described in the instructions. Use one line for each entity; add as many linesasyou need by clicking the "Add +" box.You should report relationships that were **present during the 36 months prior to publication**.

Are thereany relevant conflicts of interest? ☐ Yes ☒ No

ADD

Section 4 Intellectual Property -- Patents&Copyrights

Do you haveany patents, whether planned, pending or issued, broadly relevant to the work? ☐ Yes ☒ No

SAVE

ICMJEForm for Disclosure of Potential Conflictsof Interest

Section 5 Relationships not covered above

Are there other relationships or activities that readerscould perceive to have influenced, or that give theappearance of potentially influencing, what you wrote in thesubmitted work?

☐ Yes, the following relationships/conditions/circumstances are present (explain below):

☒ No other relationships/conditions/circumstances that present a potential conflict of interest

At the time of manuscript acceptance, journals will askauthors to confirm and, if necessary, update their disclosurestatements. On occasion, journals mayaskauthors to disclose further information about reported relationships.

Section 6 DisclosureStatement

Based on theabove disclosures, this form will automatically generatea disclosurestatement, which will appear in the box below.

Generate DisclosureStatement

Dr. Bou-Gharios has nothing to disclose.

Evaluation and Feedback

Please visit http://www.icmje.org/cgi-bin/feedback to provide feedback on your experience with completing this form.
